# Dynamic gravity compensation does not increase detection of myocardial ischemia in combined accelerometer and gyro sensor measurements

**DOI:** 10.1038/s41598-018-35630-x

**Published:** 2019-02-25

**Authors:** Magnus Reinsfelt Krogh, Per Steinar Halvorsen, Ole Jakob Elle, Jacob Bergsland, Espen Wattenberg Remme

**Affiliations:** 1 The Intervention Centre, Oslo University Hospital, Oslo, 0372 Norway; 2 The Department of Informatics, University of Oslo, Oslo, 0373 Norway; 3 The Institute for Surgical Research, Oslo University Hospital, Oslo, 0372 Norway

## Abstract

Previous studies have shown that miniaturised accelerometers can be used to monitor cardiac function and automatically detect ischemic events. However, accelerometers cannot differentiate between acceleration due to motion and acceleration due to gravity. Gravity filtering is essential for accurate integration of acceleration to yield velocity and displacement. Heart motion is cyclic and mean acceleration over time is zero. Thus, static gravity filtering is performed by subtracting mean acceleration. However, the heart rotates during the cycle, the gravity component is therefore not constant, resulting in overestimation of motion by static filtering. Accurate motion can be calculated using dynamic gravity filtering by a combined gyro and accelerometer. In an animal model, we investigated whether increased accuracy using dynamic filtering, compared to using static filtering, would enhance the ability to detect ischemia. Additionally, we investigated how well the gyro alone could detect ischemia based on the heart’s rotation. Dynamic filtering tended towards lower sensitivity and specificity, using receiver operating characteristics analysis, for ischemia-detection compared to static filtering (area under the curve (AUC): 0.83 vs 0.93, p = 0.125). The time-varying gravity component indirectly reflects the heart’s rotation. Hence, static filtering has the advantage of indirectly including rotation, which alone demonstrated excellent sensitivity to ischemia (AUC = 0.98).

## Introduction

Patients undergoing coronary artery bypass graft surgery are at risk of intra- and post-operative re-infarction due to occlusion of the bypass graft anastomoses. Myocardial ischemia is a precursor and early sign of infarction, and detecting ischemia intra- and post-operatively could potentially be used as an early warning system. Detection of ischemia by monitoring the electrocardiogram (ECG) ST-analysis, is only half as predictive as monitoring heart wall motion abnormalities with transesophageal echocardiography^1^. However, echocardiography requires the presence of an echocardiographer and is therefore not a feasible modality for continuous monitoring. In addition, the ultrasound probe cannot be left in place for a prolonged period. An alternative method for monitoring heart wall motion abnormalities is miniaturised accelerometers sutured to the heart as previously demonstrated^2–5^. A miniaturised sensor can be incorporated in the temporary pacemaker leads routinely attached during cardiac surgery. In this manner, motion monitoring can be performed during surgery, and in the critical post-operative phase, without adding to the surgical procedure. The accelerometer would have wires for external communication and powering bundled with the pacemaker lead wire. The combined system could therefore be extracted through the chest wall at the end of post-operative care, as is common practice with existing temporary pacemaker leads. Hence, a pacemaker lead with incorporated motion sensor will allow continuous motion monitoring of the cardiac function for as long as the temporary pacemaker lead is attached.

To assess heart function based on the measured heart wall motion, it is useful to calculate velocity and displacement by integrating the acceleration signal over time. However, during numerical integration, any bias or low frequency artefacts in the signal are greatly exaggerated. An accelerometer measures both the acceleration due to motion of the sensor and the acceleration caused by the earth’s gravity field, and cannot distinguish between the two. The gravity component therefore represents an artefact in the signal which has to be estimated and removed in order to accurately extract the motion. Assuming that heart motion is cyclic, i.e. starts and ends in approximately the same location, the *static* gravity component can be removed by subtracting the average acceleration during the cycle from the measurement. However, a *time varying* gravity component remains, which is caused by the heart rotating in the gravity field. This time varying gravity component often results in a 2–4 times over-estimation of the calculated displacement signal^6^.

Previously, we have shown that both the static and the time varying gravity component can be estimated and removed using a combined accelerometer and gyro sensor. The gyro measures angular velocity, from which rotation is calculated, and can therefore estimate and remove the gravity vector dynamically for all time points. Using this *dynamic* gravity compensation method gives a significantly more accurate measurement of cardiac motion^7^.

Whether the overestimation of heart motion due to the time varying gravity component has an effect on the ability to detect ischemia, has not yet been tested. We hypothesised that the increased accuracy achieved using dynamic gravity compensation would improve the ability to detect ischemia. We measured heart motion in an acute *in vivo* model, using a combined accelerometer and gyro sensor, at baseline and during three interventions: ischemia induced by occlusion of the left anterior descending artery (LAD), dobutamine infusion, and LAD occlusion during dobutamine infusion. Displacement was calculated using the two different gravity compensation methods: dynamic gravity compensation, and static gravity compensation. We also investigated how well the measured rotation from the gyro could be used to detect ischemia. Receiver operating characteristics (ROC) analyses were performed to determine the sensitivities and specificities towards ischemia based on the three different motion signals: static gravity compensated displacement, dynamic gravity compensated displacement, and rotation. To investigate the underlying differences between these methods, we performed frequency analyses of the motion traces.

## Results

**Figure 1:**
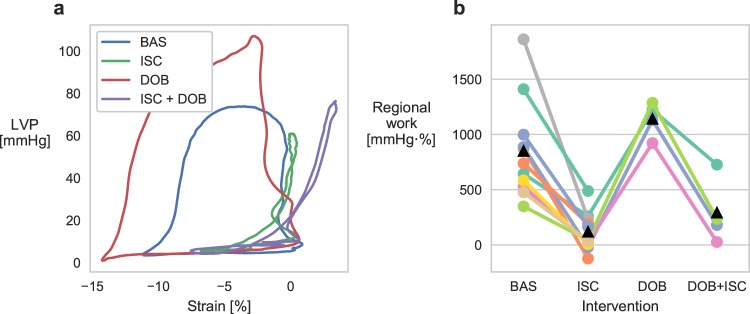
Regional myocardial work during the different interventions. (**a**) Representative LV pressure-strain loops from a segment in the anterior, apical LV region for the different interventions in one animal. (**b**) Median regional work for all animals during the different interventions: baseline (BAS), ischemia (ISC), dobutamine (DOB), and dobutamine + ischemia (DOB + ISC). Black triangles represent mean values.

Successful induction of ischemia was confirmed by regional work analysis using left ventricle (LV) pressure and sonomicrometry measurements of myocardial strain (i.e. myocardial shortening and lengthening). A typical healthy region shortens during ejection at high pressure and lengthens during filling at low pressure with relatively constant length during the isovolumic phases, hence the pressure-strain loop area resembles the pressure-volume loop. In contrast, an ischemic region typically lengthens during the isovolumic contraction phase, as a result of reduced contractility relative to surrounding regions, and shortens as pressure falls during post-systole. The pressure-strain loop area therefore decreases, reflecting less work performed by the ischemic region as shown in Fig. 1a. Fig. 1b shows that regional work was significantly reduced (*p* < 0.001, based on linear mixed model estimation with subject as random intercept) in all animals during ischemia (*n* = 11). Ischemia also reduced the regional work under dobutamine infusion, compared to dobutamine effect alone (*p* < 0.001), for all animals that underwent this intervention (*n* = 4).

### Sensitivity to ischemia based on circumferential motion

**Figure 2:**
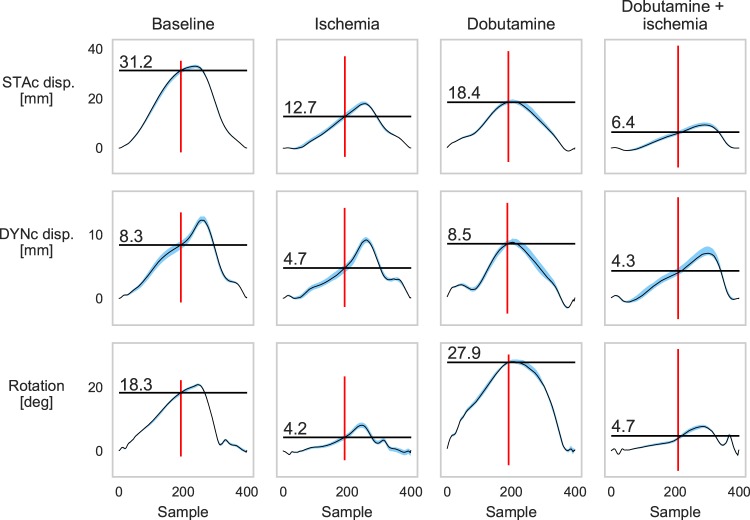
Median motion of all heart cycles for one animal during the different interventions. The duration of each cycle was normalised to 400 samples. Top row: static gravity compensated (STAc) displacement; middle row: dynamic gravity compensated (DYNc) displacement; and bottom row: rotation. Red lines represent end-systole, black horizontal lines and values represent the end-systolic motion (ESM). The blue areas represent the spread around the median (black trace) as the interquartile range. The narrow width of the blue area indicates the consistency of the motion across heart cycles.

**Figure 3:**
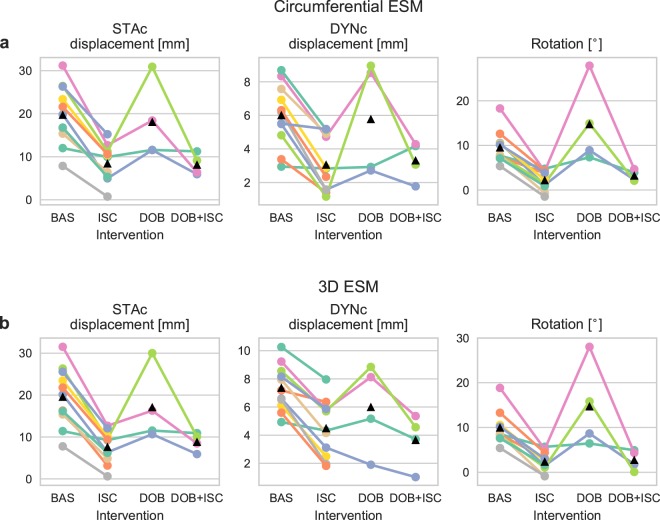
Median end-systolic motion (ESM) values per subject for (**a**) circumferential motion, and (**b**) 3D motion. Left and middle panels show displacement calculated using static (STAc) and dynamic (DYNc) gravity compensation, respectively, for each intervention (baseline (BAS), ischemia (ISC), dobutamine (DOB)). Right panels show rotation. Black triangles represent mean values.

The sensor measures motion in three spatial directions. The sensor axes were aligned parallel with the longitudinal, circumferential and radial axes of the LV. We first investigated the circumferential motion based on previous findings that circumferential motion is most affected by ischemia in the apical region where the sensor was attached^3^. To determine whether a given heartbeat was ischemic, we assessed the end-systolic motion (ESM), i.e. how far the heart had moved, or rotated, from end-diastole to end-systole. Examples of motion traces during the different interventions are shown in Fig. 2. The average circumferential ESM for static gravity compensated displacement, dynamic gravity compensated displacement, and rotation are shown in Table 1 and Fig. 3a. During ischemia, the average ESM was reduced for all three methods compared to baseline. Dobutamine infusion did not substantially increase the average ESM for neither the static gravity compensated nor the dynamic gravity compensated displacement, but there was an increase in rotation compared to baseline. Ischemia during dobutamine infusion decreased the average motion, compared to baseline, for all three methods.

**Table 1:** Observed average (and standard deviation) of hemodynamic variables and end-systolic motion (ESM) values during the different interventions for static gravity compensated (STAc) displacement, dynamic gravity compensated (DYNc) displacement, and rotation.

		Baseline (*n* = 11)	Ischemia (*n* = 11)	Dobutamine (*n* = 4)	Dob + isc (*n* = 4)
Hemodynamic variables	Regional work [mmHg·%]	850 (445)	118 (170)	1140 (159)	292 (303)
	Heart rate [bpm]	108 (21)	108 (19)	144 (36)	147 (37)
	LVP_*SYS*_ [mmHg]	82 (7)	71 (8)	100 (7)	87 (9)
	dP/dt max [mmHg s^-1^]	1086 (331)	860 (153)	2489 (618)	1810 (132)
	MAP [mmHg]	63 (16)	53 (14)	64 (14)	54 (14)
Circumferential ESM	STAc displacement [mm]	19.8 (6.9)	8.5 (4.2)	18.1 (9.1)	8.2 (2.5)
	DYNc displacement [mm]	6.0 (1.9)	3.1 (1.6)	5.8 (3.4)	3.3 (1.2)
	Rotation [°]	9.6 (3.5)	2.2 (2.1)	14.8 (9.3)	3.2 (1.3)
D ESM	STAc displacement [mm]	19.6 (7.0)	7.7 (3.8)	17.1 (8.9)	8.8 (2.2)
	DYNc displacement [mm]	7.4 (1.6)	4.5 (2.0)	6.0 (3.2)	3.7 (1.9)
	Rotation [°]	10.0 (3.6)	2.4 (2.2)	14.7 (9.7)	2.8 (2.3)

LVP_*SYS*_: left ventricular systolic pressure; MAP: mean arterial pressure.

**Figure 4:**
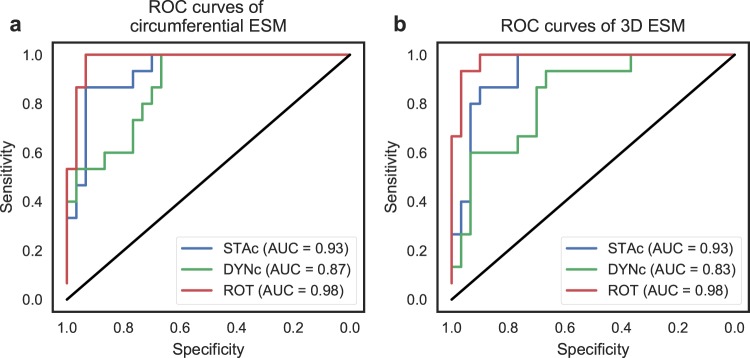
Receiver operating characteristic (ROC) curves for classifying ischemia based on end-systolic motion (ESM) from the three different signals (static gravity compensated (STAc) displacement, dynamic gravity compensated (DYNc) displacement, and rotation (ROT)), for (**a**) circumferential and (**b**) 3D motion. AUC: Area under the ROC curve.

From the results of the ROC analysis, shown in Fig. 4a, the area under the curve (AUC), i.e. the overall ischemia classification, tended towards a higher score for rotation compared to both static gravity compensated (*p* = 0.114, using DeLong’s test for comparing ROC curves^8^) and dynamic gravity compensated displacement (*p* = 0.047). There was no difference in AUC between static and dynamic gravity compensated displacements (*p* = 0.263). The optimal cutoff value for these ROC curves, chosen by requiring a specificity of 0.8, yielded a sensitivity of 0.87, 0.6, and 1.0 for static gravity compensated displacement, dynamic gravity compensated displacement, and rotation, respectively.

### Sensitivity to ischemia based on 3D motion

In a clinical setting it would be more convenient if the sensor could be placed in an arbitrary orientation rather than requiring the surgeon to align its axes parallel to the cardiac axes. It is therefore necessary to reduce the three dimensional motion measured by the sensor to a one dimensional motion trace. We applied a previously introduced algorithm^9^ that calculates the motion which travels along a 3D vector representing the main contraction direction. The ESM based on the 3D motion exhibited similar tendencies to the circumferential motion, as shown in Table 1 and Fig. 3b.

Consequently, the ROC AUC score, shown in Fig. 4b, displayed similar tendencies as with circumferential motion. The AUC for rotation was borderline significantly higher than for static gravity compensated displacement (*p* = 0.076) and significantly higher than dynamic gravity compensated displacement (*p* = 0.018). The AUC for dynamic gravity compensated displacement, in turn, tended to be lower than for static gravity compensated displacement (*p* = 0.125). The optimal cutoff value for these ROC curves, requiring a specificity of 0.8, yielded a sensitivity of 0.87, 0.6, and 1.0 for static gravity compensated displacement, dynamic gravity compensated displacement, and rotation, respectively.

### Frequency analysis

**Figure 5:**
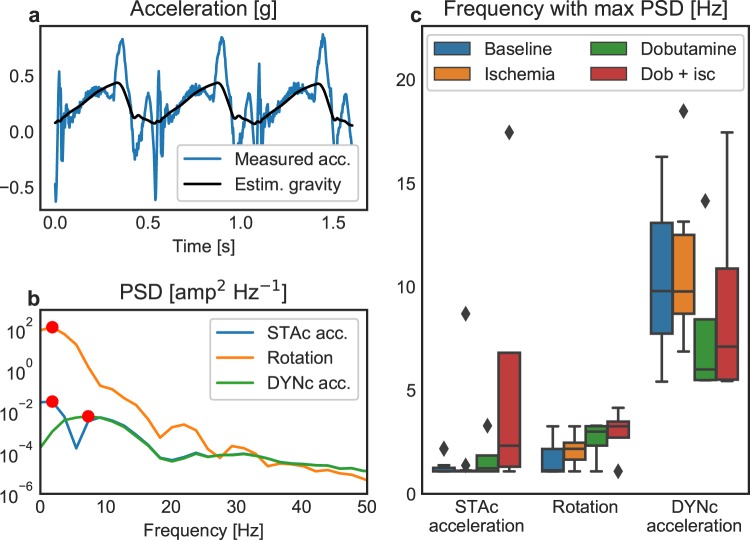
Frequency analysis of measured heart motion. (**a**) Measured acceleration in the circumferential direction over three consecutive heart cycles. This acceleration includes a gravity component that varies over the cycle with the rotation of the heart. Also shown is the gravity component, estimated using the gyro. (**b**) Power spectral density (PSD) of the signals showing that the low frequency information due to rotation is maintained in the static gravity compensated (STAc) acceleration while it is removed from the dynamic gravity compensated (DYNc) acceleration. Red circles represent the frequency containing the highest power. (**c**) Boxplot showing the frequency containing the highest power within the signals, separated by intervention.

Figure 5a shows the measured raw acceleration in the circumferential direction and the extracted time varying gravity component caused by rotation of the heart in the gravity field. Rotation, and hence the time-varying gravity component, varied slowly and most of its power was in the lower frequency bands in contrast to the more rapidly varying acceleration signal, which had more power at higher frequencies (Fig. 5b). Consequently, subtraction of the time-varying gravity component resulted in attenuated power in the lower frequency band for the dynamic gravity compensated signal. As shown in Fig. 5c, the static compensated acceleration signal had, on average, most of its power at 2.0 Hz, which was the same as for the measured rotation (2.0 Hz), which was directly related to the heart rate as the apical region, when viewed from the apex, rotates counter-clockwise during systole and clockwise during diastole in a sine wave like motion. The dynamic compensated acceleration signal had the majority of power in a higher frequency band, with an average of 9.9 Hz. This demonstrates that information of rotation was still incorporated in the static compensated signal, while it was removed from the dynamic compensated signal.

## Discussion

This study shows that adding a gyro to the accelerometer in order to improve accuracy of calculated systolic displacement^7^ did not result in improved detection of ischemia compared to displacement calculated from pure accelerometer measurements. On the contrary, using dynamic gravity compensation by gyro tended to decrease sensitivity and specificity towards detecting ischemia, as it removed the indirect measure of the heart’s rotation reflected in the time-varying rotational gravity component.

The difference between static and dynamic gravity compensation can be investigated in the frequency domain in order to disclose this result (Fig. 5). Displacement is the double integral of acceleration. Double integration suppresses high frequencies and amplifies low frequencies in the signal, equivalent to multiplying the frequency spectrum by a factor of 1/*frequency*^2^. Hence, acceleration contains higher temporal oscillations and more of its power is at higher frequencies. The gravity component in the measured acceleration is not constant along an axis, but varies slowly over the heart cycle as the axis rotate in the gravity field (Fig. 5a). Therefore, accurate gravity filtering requires a dynamic gravity compensation. In contrast, static compensation (i.e. subtracting the mean gravity component over one heart cycle) leaves a slow varying component linked to the rotation. The static compensated acceleration thus includes this low frequency component linked to rotation, which is then further amplified by the double integration to displacement. Our results show that rotation is highly sensitive to ischemia, which is in accordance with results from previous studies which have used other methods^10–14^. Thus, removing all information of rotation by dynamic compensation also reduced its sensitivity towards detecting ischemia compared to static compensation. On the other hand, the added value of gravitational rotation in the measurement comes with limitations: the dynamic gravity component is proportional to the inclination of the heart’s long axis relative to the horizontal plane^6^. As an example, if measuring on a patient in the supine position, then the plane of rotation of the heart will be more or less perpendicular to the horizontal plane. In this case the rotational component will be at its maximum. On the other hand, if the patient’s thorax is upright, the rotation of the heart would be parallel to the horizontal plane, and there would be close to no rotational gravity component. However, in the clinic, evaluation of ischemia by this method will most likely occur, or can easily be performed, when the patient lies in the standard supine position. Hence, evaluation when the patient is sitting or moving might be of limited interest or not of critical importance. However, if cardiac accelerometer measurements are going to be used on patients which are not in a supine position, this limitation needs to be taken into account.

Assessing the measured rotation, using gyro alone, yielded the best sensitivity and specificity to ischemia. This is reflected both in the excellent ROC AUC scores, which were significantly higher than for dynamic gravity compensated displacement, and tended to be higher than for static gravity compensated displacement, in both circumferential and 3D motion. Using a gyro sensor for the purpose of detecting ischemia has also previously been suggested^13–15^. Gyro sensors have the advantage of not being affected by gravity, since these sensors measure angular velocity and not acceleration. Additionally, gyro sensors are less susceptible to low frequency bias artefacts as they require only single integration to yield rotation, whereas accelerometers require double integration to yield displacement. However, at present, the smallest physical size of a gyro sensor is, to our knowledge, 3 × 2.5 × 0.83 mm (BMG250, Bosch Sensortec GmbH, Kusterdingen, Germany), which is about 3 times larger than the size of the smallest accelerometer (1.29 × 1.09 × 0.74 mm, MC3672. mCube, Inc, San Jose, CA, USA). In a potential product where the sensor is placed epicardially, the wire attached to the sensor must be tunnelled, and eventually extracted, through the chest wall, similar to temporary pacemaker leads. The size of the sensor therefore plays a vital role in the product’s clinical feasibility, and until the size of gyro sensors becomes smaller, it is more clinically feasible to use accelerometers.

Our study was performed in an *in vivo* animal model. Advantageously, since these experiments were acute, we were able to perform highly invasive measurements which had not been feasible in patients, e.g. left ventricle pressure measurements and regional strain measurements using sonomicrometry. On the other hand, the effect of LAD occlusion in healthy animals may be different from the effect in patients since ischemia in a patient is not only dependent on graft patency, but also on the presence of collateral blood supply and flow through the native vessel. This might be reflected in an overly confident ischemia detection score. However, this study was intended to investigate whether there were differences in the ability to detect ischemia between using static gravity compensated displacement, dynamic gravity compensated displacement, and rotation, and not a clinical test of the accuracy. Sensitivity of static gravity compensated motion measurements has previously been reported^4^ with scores comparable to what we have shown in this study.

One limitation of this study is that we only assessed regional ischemia due to LAD occlusion, whereas other myocardial regions would have been affected by ischemia due to right coronary artery (RCA) occlusion, or circumflex artery (CX) occlusion. Although myocardial strain in remote, healthy regions is not directly affected by myocardial dysfunction occurring in another region, the motion of the myocardial tissue of healthy regions is affected as they are tethered to the dysfunctional region^16^. The global motion pattern is therefore affected by regional dysfunction. For the purpose of early detection of ischemia, measuring a global effect is advantageous as this suggests that only one sensor is sufficient. Previous studies have shown that accelerometers placed in the regions supplied by CX^4,9^ and RCA^17^ are affected by LAD occlusion, although to lesser degree. However, whether the reverse is true, i.e. occlusion of RCA and CX affects an accelerometer placed in the apical region, is unknown. The effect of CX and RCA occlusions on accelerometer and gyro measurements should therefore be investigated in future studies.

We have in this study shown that adding a gyro to perform time varying gravity compensation of cardiac accelerometer measurements, does not improve the ability to detect ischemia. This is despite dynamic gravity compensation yielding higher accuracy of the measured heart motion; however, it is because the time varying gravity component is an indirect measure of the heart’s rotation, and the heart’s rotation is highly sensitive to ischemia. Removing this gravity component therefore reduces the sensitivity of the accelerometer measurement towards detecting ischemia. Our results also show that measuring the rotation of the heart, using a gyro sensor alone, gives better sensitivity and specificity towards detection of ischemia.

## Methods

### Sensor design

**Figure 6:**
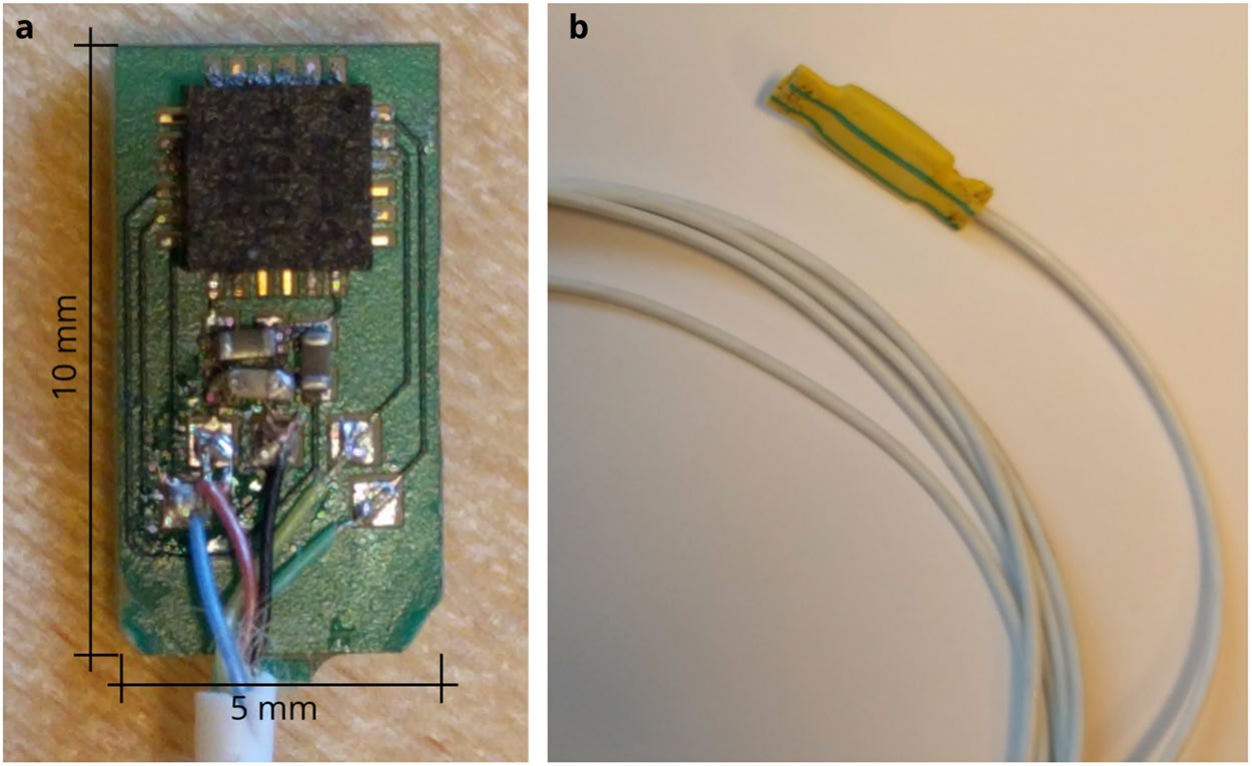
Sensor design. (**a**) Picture showing the assembled circuit board of the motion sensor without coating. (**b**) Picture showing the complete sensor with plastic cover and cable.

The prototype sensor package consisted of a combined accelerometer and gyro motion sensor (MPU9250, Invensense, Inc., San Jose, CA, USA), with a sampling rate of 700 S/s, placed on a small circuit board (10 × 5 mm), enclosed in epoxy, shown in Fig. 6a. The accelerometer part measures the changes in displacement of a proof mass suspended within capacitive sensors which is proportional to the acceleration of the mass. The gyro part measures the rate of rotation as the Coriolis effect force exerted on a vibrating proof mass using capacitive sensors. A thin, flexible wire, mounted to the circuit board, connected the sensor to a data acquisition unit. The sensor package was enclosed in a shrinking tube of material which could be perforated for suturing to the heart, as shown in Fig. 6b.

We used a data acquisition unit (Cardiaccs AS, Oslo, Norway) that communicated with the sensor, which in addition simultaneously recorded an ECG signal. We developed a driver software that gathered the raw data from the sensor and ECG, and forwarded it to a data recording application. The data recording application was implemented using LabView (National Instruments, Austin, TX, USA).

### Experiment setup

#### Animal preparation

**Figure 7:**
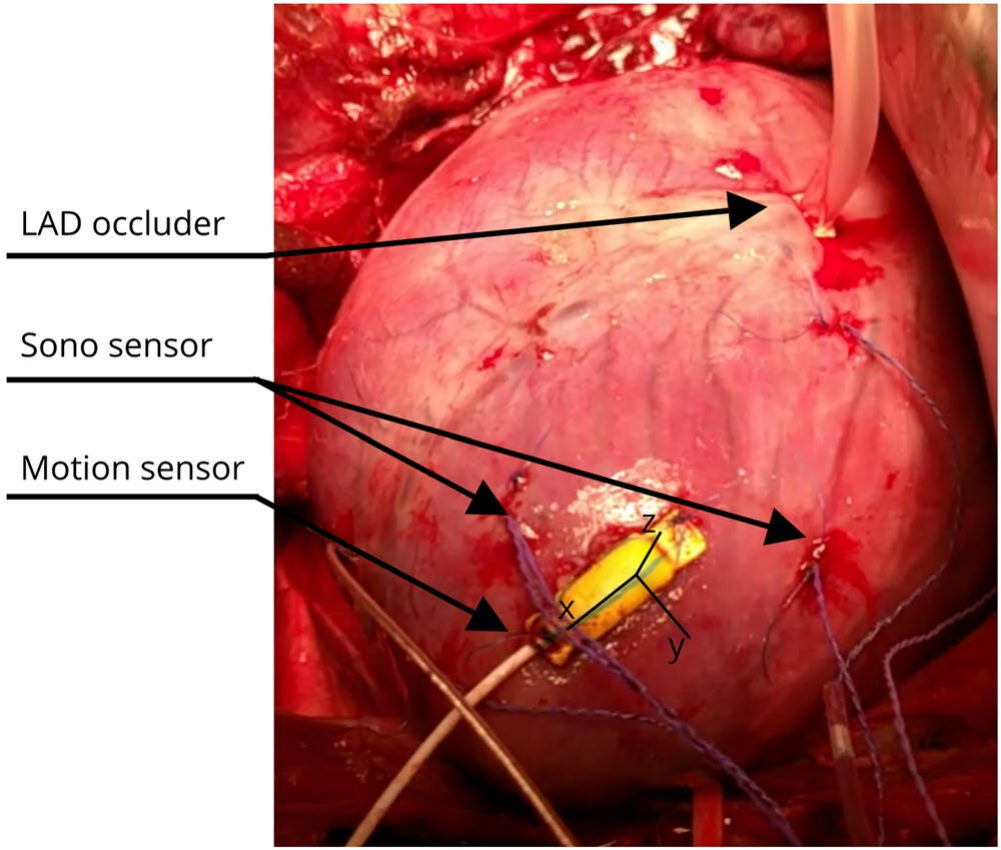
Picture of the heart after instrumentation in animals. The motion sensor was placed in the LV apical region, parallel to the long axis. Black lines represent the coordinate system of the motion sensor. Sonometrics sensors were placed on either side of the motion sensor. A snare was placed proximally around the left anterior descending artery (LAD).

The animal study was approved by the National Animal Experimentation Board [project ID: 9303], and carried out in accordance with Norwegian regulations concerning use of animals in experiments [FOR-2015-06-18-761]. Fifteen NOROC pigs of either sex and average body weight 44 ± 2 kg (±s.d.) were anaesthetised and prepared as previously described in detail^4^. In short: after anaesthesia was induced, a median sternotomy was performed exposing the pericardium. Pericardium was split from base to apex exposing the heart. We then sutured the motion sensor to the LV apical region, parallel to the longitudinal axis, as shown in Fig. 7. Micromanometer catheters (Millar, Inc., Houston, TX, USA) were inserted through introducers in the carotid arteries and placed in the LV and ascending aorta for continuous measurement of the LV and aortic pressures. Sonomicrometry sensors (Sonometrics, Corp., London, ON, Canada), the gold standard for measuring segment lengths in tissue, were placed on either side of the motion sensor. These sensors were placed along the subendocardial muscle fibre direction. Lastly, we placed a snare around the LAD as proximal as possible, as shown in Fig. 7.

#### Experiment protocol

Data were recorded during baseline (*n* = 11), LAD occlusion (after 76 ± 44 s (±s.d.) acute occlusion (*n* = 9), or after 549 ± 66 s (±s.d.) gradual occlusion (*n* = 2)), dobutamine infusion (*n* = 4), and LAD occlusion during dobutamine infusion (*n* = 4).

#### Limitations regarding the experimental setup

A total of 15 experiments were conducted. Three experiments were excluded due to fatal complication in the surgery before any data were gathered. One pilot experiment was excluded due to technical issues. In 8 of the successful experiments, the animals were first subject to loading and unloading of blood volume, as part of another experiment protocol. As part of this other protocol we performed additional instrumentation including placement of a pulmonary artery catheter (Edwards Lifesciences Corporation, Irvine, CA, USA) through an introducer in the right jugular vein, PiCCO catheter (PV 2025L20, PULSIOCATH; Pulsion Medical Systems, Munich, Germany) inserted in the femoral artery, and four additional sonomicrometry sensors placed in the apex, base, anterior, and posterior regions of the LV. In 2 of the pilot experiments the flow of blood supply to the LAD was controlled by occluding the LAD and bypassing it with the left internal mammary artery. The amount of flow going to the LAD was then controlled by an adjustable occluder and the blood flow was measured using transit time flowmetry (Transsonic Systems Inc. Ithaca, NY, USA). Ischemia in these two experiments were gradually induced over several minutes.

### Data analysis

All signal processing and analyses were performed using Python 3.6. From the measurements we used data covering at least three respiration cycles. ECG R-peaks were automatically determined using the Python package BioSPPy^18^. The R-peak markers were then manually checked and corrected.

Static gravity compensated displacement was calculated as the second integral of acceleration, after static gravity was removed by subtracting the average acceleration. Dynamic gravity compensated displacement was calculated from acceleration after dynamic gravity was removed from the measurement, using the previously described algorithm^7^. Rotation measurements were calculated as the integral of angular velocity.

From the sonometrics sensors we calculated regional strain, and together with the LV pressure we calculated an index of regional work as the area of the LV pressure-strain loop. This measurement was used to determine if ischemia was successfully induced.

As motion in the circumferential direction is highly sensitive to ischemia^5^, the sensor was placed parallel to the longitudinal axis. In this manner the sensor’s y-axis pointed in the circumferential direction and motion along the circumferential axis could be extracted and analysed directly. Additionally, we analysed the 3D sensor motion by extracting the main contraction direction relative to the sensor’s coordinate system using a previously described algorithm^9^. In short: the main contraction direction was calculated at baseline as the vector between the position of the sensor at the start of the heart cycle, to the point where it was farthest away from the starting point during the cycle. The effects of the different interventions were then assessed as the motion trace resulting from projecting the measured motion onto this reference vector.

To determine ischemia we assessed the motion at ES as shown in Fig. 2. We measured invasive LVP and could therefore determine the actual time point of ES as *dP*/*dt*_*min*_^19^. However, to reflect a clinical situation, where invasive LVP measurements would not be available, we chose to estimate this time point. Boudoulas *et al*.^20^ has previously shown that there is a linear relationship between the length of electromechanical systole (QS_2_, Q: ECG Q-wave, S_2_: second heart sound) and heart rate (HR). This relationship was based on humans with resting heart rates between 40 and 110 bpm. Since we used pigs, with a somewhat different electrophysiology, and with a range of heart rates from 80 to 200 bpm, this linear model would not necessarily be transferable to our animals. We found that the Boudoulas method had an average error of 8 ± 28 ms (±s.d.) which was 5 ± 5% (±s.d.) of the total cycle length. Additionally, we defined the start of the systole as the ECG R-peak, and not the Q-wave, and therefore had to estimate the duration R-*dP*/*dt*_*min*_, rather than QS_2_. We therefore derived a regression model between the duration R-*dP*/*dt*_*min*_ and heart rate, and found that this relationship was curvilinear: ${t}_{R\to dP/d{t}_{{\min }}}=0.61-4.317\,\cdot \,{10}^{-3}HR+1.05\,\cdot \,{10}^{-5}H{R}^{2}$ (*r*^2^ = 0.79, *p* < 0.001). This formula gave an average error of 3 ± 22 ms (±s.d.) which was 2 ± 2% (±s.d.) of the total cycle length, and was used to define ES in our measurements. In a clinical application, however, the estimation of ES has to be determined using a method more suited to humans, like the previously mentioned method suggested by Boudoulas *et al*.

To analyse the sensitivity and specificity of the ischemia metric, we performed ROC analyses. Ischemia with and without dobutamine were grouped together as positive. Baseline and dobutamine, plus their respective reperfusions, were grouped together as negative. Although this gives a skewness in the distribution of negative vs positive samples of 2:1, one inherent property of ROC analysis is insensitivity to class distribution skewness^21^. To account for the correlation in the measurements, we first modelled the response, i.e. probability of ischemia, as a logistic mixed model with subject as random intercept, and the respective ESM values as explanatory variables. The predicted outcome from this model was then used as input to the ROC analysis. The area under the ROC curve was used as overall ischemia detection score for comparison between static gravity compensated displacement, dynamic gravity compensated displacement, and rotation.

To analyse the frequency components of the raw acceleration, dynamic gravity compensated acceleration, and rotation, the power spectral density for each measurement was calculated using fast Fourier transform with a window size of 600 samples (595 samples overlap). This gave a proportional distribution of the power for the frequency components in the signal, which was used to compare the signals.

### Statistics

All statistical calculations were performed using R v3.4.1. The results in this study were calculated from repeated, correlated measures within each subject. To account for the correlations between measurements, and the missing data in the dobutamine intervention, we used linear mixed models with subject as random intercept to model the differences between the interventions. To test differences between individual estimates, we performed linear combinations statistical tests. The ROC curves were calculated using the R package pROC^22^. To test statistical difference between two correlated ROC curves we used DeLong’s test^8^. Significance level was set at *p* < 0.05.

## References

[1] Comunale, M. E. *et al*. The concordance of intraoperative left ventricular wall-motion abnormalities and electrocardio-graphic s-t segment changes: Association with outcome after coronary revascularization. *Survey of Anesthesiology* **43**, 197–198, https://journals.lww.com/surveyanesthesiology/Fulltext/1999/08000/The_Concordance_of_Intraoperative_Left_Ventricular.10.aspx (1999). 10.1097/00000542-199804000-00014 9579503

[2] Elle OJ (2005). Early recognition of regional cardiac ischemia using a 3-axis accelerometer sensor. Physiological Measurement.

[3] Halvorsen PS (2008). Feasibility of a three-axis epicardial accelerometer in detecting myocardial ischemia in cardiac surgical patients. The Journal of Thoracic and Cardiovascular Surgery.

[4] Halvorsen PS (2009). Detection of myocardial ischaemia by epicardial accelerometers in the pig. BJA: British Journal of Anaesthesia.

[5] Halvorsen PS (2010). Automatic real-time detection of myocardial ischemia by epicardial accelerometer. The Journal of Thoracic and Cardiovascular Surgery.

[6] Remme EW (2009). Validation of cardiac accelerometer sensor measurements. Physiological Measurement.

[7] Krogh MR (2017). Gravity compensation method for combined accelerometer and gyro sensors used in cardiac motion measurements. Annals of Biomedical Engineering.

[8] DeLong, E. R., DeLong, D. M. & Clarke-Pearson, D. L. Comparing the areas under two or more correlated receiver operating characteristic curves: A nonparametric approach. *Biometrics* **44**, 837–845, http://www.jstor.org/stable/2531595 (1988). 3203132

[9] Grymyr O-JH (2015). Assessment of 3d motion increases the applicability of accelerometers for monitoring left ventricular function†. Interactive CardioVascular and Thoracic Surgery.

[10] Helle-Valle T (2005). New noninvasive method for assessment of left ventricular rotation: speckle tracking echocardiography. Circulation.

[11] Govind SC, Gadiyaram VK, Quintana M, Ramesh SS, Saha S (2010). Study of left ventricular rotation and torsion in the acute phase of st-elevation myocardial infarction by speckle tracking echocardiography. Echocardiography.

[12] Sun JP (2007). Alterations of regional myocardial function in a swine model of myocardial infarction assessed by echocardiographic 2-dimensional strain imaging. Journal of the American Society of Echocardiography.

[13] Cercenelli L, Marcelli E (2015). Cardiac apex rotation assessed by an implantable gyro sensor: correlation with a lv pressure-derived myocardial performance index in experimentally induced ischemia. Journal of Mechanics in Medicine and Biology.

[14] Marcelli, E. *et al*. Assessment of cardiac rotation by means of gyroscopic sensors. In *2008 Computers in Cardiology*, 389–392, 10.1109/CIC.2008.4749060 (2008).

[15] Marcelli, E., Plicchi, G., Cercenelli, L. & Bortolami, F. First experimental evaluation of cardiac apex rotation with an epicardial coriolis force sensor. *ASAIO Journal* **51**, 696–701, https://journals.lww.com/asaiojournal/Fulltext/2005/11000/First_Experimental_Evaluation_of_Cardiac_Apex.18.aspx (2005). 10.1097/01.mat.0000179250.52117.5c 16340353

[16] Skulstad H (2006). Grading of myocardial dysfunction by tissue doppler echocardiography. Journal of the American College of Cardiology.

[17] Grymyr O-JH (2015). Continuous monitoring of cardiac function by 3-dimensional accelerometers in a closed-chest pig model†. Interactive CardioVascular and Thoracic Surgery.

[18] Carreiras, C. *et al*. BioSPPy: Biosignal processing in Python (2015–). https://github.com/PIA-Group/BioSPPy/ (Online; accessed January 2018).

[19] Abel FL (1981). Maximal negative dp/dt as an indicator of end of systole. American Journal of Physiology-Heart and Circulatory Physiology.

[20] Boudoulas H, Geleris P, Lewis RP, Rittgers SE (1981). Linear relationship between electrical systole, mechanical systole, and heart rate. CHEST.

[21] Fawcett T (2006). An introduction to roc analysis. Pattern Recognition Letters.

[22] Robin X (2011). proc: an open-source package for r and s+ to analyze and compare roc curves. BMC Bioinformatics.

